# Study on the Migration Patterns of Oxygen Elements during the Refining Process of Ti-48Al Scrap under Electromagnetic Levitation

**DOI:** 10.3390/ma17153709

**Published:** 2024-07-26

**Authors:** Xinchen Pang, Guifang Zhang, Peng Yan, Zhixiang Xiao, Xiaoliang Wang

**Affiliations:** Faculty of Metallurgical and Energy Engineering, Kunming University of Science and Technology, Kunming 650093, China; xinchen001118@163.com (X.P.); xiaozhixiang1998@163.com (Z.X.); wangxiaoliang@kust.edu.cn (X.W.)

**Keywords:** electromagnetic levitation, Ti-Al alloy scrap, refining deoxidation, elemental oxygen migration

## Abstract

This study investigated the migration patterns of oxygen in the deoxidation process of Ti-48Al alloy scrap using electromagnetic levitation (EML) technology. Scanning electron microscopy (SEM), X-ray diffraction (XRD), and X-ray photoelectron spectroscopy (XPS) were employed to analyze the oxygen distribution patterns and migration path during EML. The refining process resulted in three types of oxygen migration: (1) escape from the lattice and evaporation in the form of AlO, Al_2_O; (2) formation of metal oxides and remaining in the alloy melt; (3) attachment to the quartz tube wall in the form of metal oxides such as Al_2_O_3_ and Cr_2_O_3_. The oxygen content of the scrap was dropped with a deoxidation ratio of 62%. It indicated that EML can greatly promote the migration and removal of oxygen elements in Ti-Al alloy scrap.

## 1. Introduction

Titanium alloys are widely utilized in the chemical and aerospace industries, marine engineering, and a variety of other areas owing to their excellent characteristics, such as low weight, high specific strength, high temperature resistance, corrosion resistance, and outstanding antioxidant properties [1,2,3]. Particularly, titanium–aluminum (Ti-Al) alloys are considered to have great application prospects in the development of high-temperature structural materials for aerospace, defense, and automotive industries [4]. They need to withstand harsh operating conditions, including high temperatures, severe air friction, and cyclic oxidation [5], leading to a large amount of oxidized scrap during melting, processing, and subsequent operations. Due to its high affinity for Ti [6], oxygen tends to dissolve preferentially in the α phase of Ti alloys, thereby strengthening this phase [7]. In particular, the solubility limit of oxygen can reach 14 wt.% in the hexagonal α-Ti phase [8], making it extremely challenging to deoxidize from titanium scrap. The ideal and most economical method of utilizing Ti scrap is to melt it back into ingots. However, it is essential to reduce the oxygen content of titanium scrap before returning to smelting. Thus, investigations about simple and effective deoxidation methods for Ti-Al alloys and Ti alloy scrap are highly focused. At present, the deoxygenation techniques for Ti scrap include molten-salt electrochemical deoxidation [9], hydrogenation–dehydrogenation (HDH) [10], and the smelting deoxidation process [11,12]. Suzuki et al. [13,14] have explored the molten-salt electrolytic deoxidation method. Metallic Ca is obtained by electrolyzing CaO in molten salt, followed by calciothermically reducing TiO_2_ to form sponge Ti. This method has a lot of advantages, such as low process costs, minimal energy consumption, and high purity of the Ti sponge product. On the other hand, parasitic reactions result in a decrease in current efficiency, such as carbon precipitation during electrolysis. Then, the separation of the product from the electrolyte becomes challenging due to the structural issues in the electrolytic cell [15].

Su et al. [16] have used the HDH process to melt the Ti64 alloy, reducing its oxygen content from 0.12 to 0.028 wt.%. This process involves hydrogen atoms entering the lattice to form metal hydrides, followed by dehydrogenation under appropriate experimental conditions to yield Ti powder [17]. Although this method involves a short process flow and simple equipment, it presents difficulties in the complete removal of hydrogen during the dehydrogenation process, particularly for the scrap with a high oxygen content [18]. Bartosinski et al. [19,20] utilized a vacuum induction melting furnace to investigate the preparation of Ti-6Al-4V by aluminothermic reduction. This method resulted in a decrease in the oxygen content by 1500–3500 ppm and significantly lowered the production cost of Ti alloys. Despite these advancements, these challenges have to be overcome for the utilization of Ti scrap; for example, high oxygen content and the presence of numerous inclusions persist. Electromagnetic levitation (EML) technology, characterized by rapid surface renewal and vigorous internal stirring [21,22], can offer favorable kinetic conditions for volatilization and impurity removal, which can lead to an ideal metallurgical reaction process.

This study proposes the utilization of EML melting technology for deoxidation from titanium scrap, capitalizing on its advantages in strengthening metallurgy. To address the current limitations of conventional melting and deoxidation processes, such as sluggish diffusion of internal solute elements and restricted surface renewal frequency, the elemental migration process during the intensified deoxidation process was focused on in this study. Finally, the results from this work should aid in future adoptions of shorter and cost-effective processes in the utilization of oxygen-containing Ti scrap.

## 2. Materials and Methods

### 2.1. Materials

The raw material for this study is titanium scrap produced during the production processes of a factory, with Al scrap, Cr scrap, and TiO_2_ powder added. The prepared alloy was tested for its composition as Ti-48Al-2Cr (Ti: Al: Cr = 50: 48: 2 at.%; O 0.5 at.%). The Ti-48Al scrap was cut into samples weighing approximately 1.2 g each for the EML experiments. The argon gas used in the experiments was supplied by Guangruida Gas Co., Ltd., Kunming, China, with a purity of 99.999%.

### 2.2. Method

**Figure 1:**
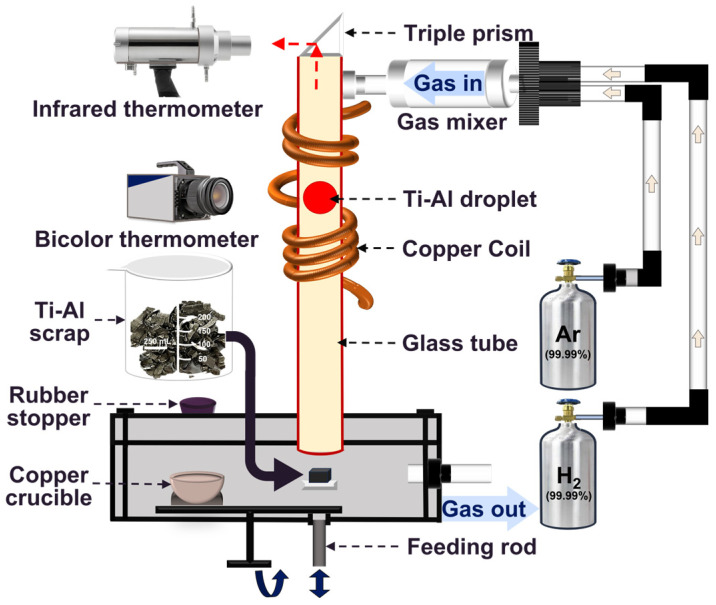
Schematic depiction of the EML system.

The procedure and experimental setup for the EML experiment are depicted in Figure 1. The equipment mainly consists of an electromagnetic levitation melting system, atmosphere control system, and camera detection system. Before activating the device, a stream of pure argon gas was supplied at a rate of 1.5 L/min (liters per minute) to remove air presented in the glass tube. The deoxygenation method performed on the Ti-Al alloy scrap using EML has three stages: preheating, levitation melting, and levitation refining. At the end of the refining process, the power current was gradually reduced to below 0.7 A and the melt precipitated and fell into the copper crucible and solidified. Afterwards, the power supply was turned off, and bubbled Ar gas was used as a cooling atmosphere until the scrap approached room temperature. To ensure the reliability of the experimental data, three repetitions of the experiment were carried out, and the elemental oxygen content was tested.

X-ray Diffraction (X’pert 3, Malvern-Panalytical Powder) was used to analyze the phase composition of the samples before and after EML. The variation in oxygen content was identified using an oxygen/nitrogen analyzer (HORIBA EMGA-830, Kyoto, Japan). The microstructure and element composition of the samples was detected using a scanning electron microscope (HITACHI SU8010, Tokyo, Japan) and via energy-dispersive X-ray spectroscopy (OXFORD ULTIM MAX400, Oxford, UK). Additionally, X-ray photoelectron spectroscopy (pHI5000 VERSAPROB-II, Tokyo, Japan) was used to study the changes in the valence states of oxygen and other elements during the removal process under EML.

## 3. Results and Discussion

### 3.1. Thermodynamic Analysis of Deoxygenation

The volatilization behavior of the Ti-Al alloys was investigated through the calculation of saturated vapor pressures of Ti, Al, and Cr at different temperatures using the Clausius–Clapeyron equation (C-C equation) [23]:

(1) $$logP_{i}^{*}=AT^{-1}+BlogT+CT+D$$

where *T* (K) represents thermodynamic temperature, $P_{i}^{*}$ denotes the saturated vapor pressure (Pa) of ideal metal at temperature *T*. The fixed values in the C-C equation at 2173 K are shown in Table 1.

**Table 1:** The C-C equation parameters for Ti, Al, and Cr in Ti-Al alloys at 2173 K [24,25].

Element	A	B	C	D
Ti	−24,914	−2.52	—	20.832
Al	−16,450	−1.023	—	14.48
Cr	−20,680	−1.31	—	16.68

**Figure 2:**
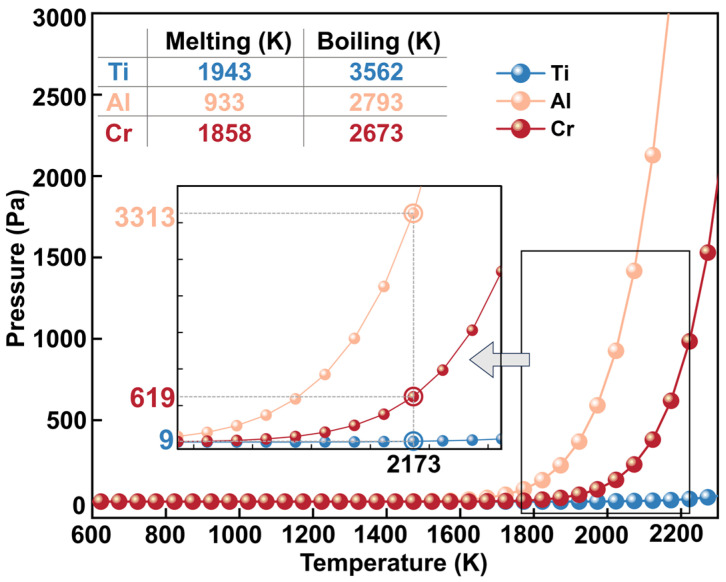
Temperature variation in saturation vapor pressure of Ti, Al, and Cr in Ti-Al alloys.

Figure 2 depicts the variation in saturation vapor pressure with temperature for the elements Ti, Al, and Cr in the Ti-Al alloys. At temperatures ranging from 0 to 1600 K, all the curves are approximately the same and converge to 0, suggesting that the Al, Ti, and Cr components are not easily volatilized under melting conditions of lower temperature. When the temperature exceeds 1600 K, the saturated vapor pressure of Al and Cr starts to increase exponentially, while Ti remains relatively constant; when it rises to 2173 K, the saturated vapor pressure of Al reaches 3313 Pa at this time, and Cr also reaches 619 Pa, while the Ti element remains at around 9 Pa. Ono et al. [26] categorized refractory metals based on the difficulty of deoxygenation, grouping them from I to IV according to the level of difficulty. The saturation vapor pressures of Ti and Ti oxides in group IV at a temperature of 1800 K are 5.2 × 10^−3^ Pa and 1.3 × 10^−2^ Pa, respectively. Therefore, it is highly unlikely for elemental oxygen to be removed through evaporation by combining it with Ti. Under identical thermal conditions, Cr and its oxides in group II have saturation vapor pressures of 9.6 Pa and 4.0 × 10^−3^ Pa, respectively. Metals in this group have low oxygen solubility and activity; deoxygenation is effective when Cr content is above a certain amount. However, the Cr content in the alloy matrix is much lower than that of the Ti and Al in this study; so, the impact of its volatilization on the oxygen content is currently not being taken into account.

Based on the relevant literature [27], two main forms in which oxygen can be removed through evaporation reactions in Ti-Al system are as follows:

(2) $$Al\left(l\right)\left(in\;molten\;TiAl\right)+O\left(in\;molten\;TiAl\right)\to AlO(g)↑$$

(3) $$2Al\left(l\right)\left(in\;molten\;TiAl\right)+O\left(in\;molten\;TiAl\right)\to {Al}_{2}O(g)↑$$

This study covers a temperature range from 1973 to 2273 K [28,29]; the Gibbs free energy remains consistently negative and increases in magnitude with rising temperature. The result suggests that oxygen can be removed through the volatilization of Al in Ti-Al system, with reactions occurring more easily at higher temperatures.

### 3.2. Results of Deoxidation Experiments under Electromagnetic Levitation Conditions

**Figure 3:**
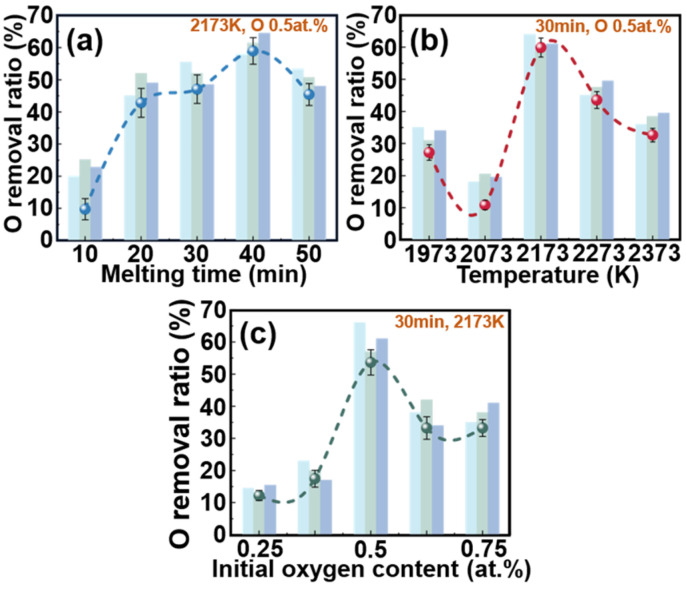
Results of deoxidation experiments under different electromagnetic levitation conditions: (**a**) melting time, (**b**) temperature, and (**c**) initial oxygen content.

The transfer behavior of impurity elements in the electromagnetic levitation refining process is significantly influenced by various EML conditions. Consequently, this study aimed to examine the effect of different melting time, temperature, and initial oxygen content on the removal efficiency of oxygen in Ti-Al alloys; the results are depicted in Figure 3. As depicted in Figure 3a, under the five melting times of 10 min, 20 min, 30 min, 40 min, and 50 min, the oxygen removal rate increases gradually from 10 min to 40 min and then decreases with longer melting times. The oxygen removal ratio at 40 min is 61.1%, significantly higher than at other melting times. Therefore, a levitation time of 40 min is suitable for the further experiments. Subsequently, as shown in Figure 3b, the experiments were conducted at five temperatures in the range of 1973–2373 K, with a levitation melting temperature of 2173 K exhibiting a remarkably high oxygen removal ratio of 61.3%. Ultimately, the experiments with varying initial oxygen contents were conducted at a temperature of 2173 K and levitation time of 40 min. The oxygen contents ranged from 0.25 at.% to 0.75 at.%, as illustrated in Figure 3c, which revealed that the highest oxygen removal ratio occurred at an initial oxygen content of 0.5 at.%. Consequently, it was determined that the optimal levitation parameters for a 61.3% deoxidation ratio are a temperature of 2173 K, levitation time of 40 min, and initial oxygen content of 0.5 at.%.

### 3.3. Results of the Changes in Elemental Distribution and Phase Composition during EML Experiments

**Figure 4:**
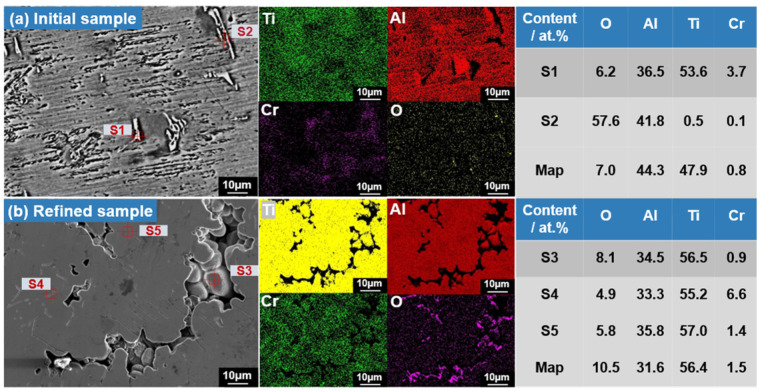
The EDS detection images of the Ti-Al alloy (**a**) before EML and (**b**) under conditions of 2173 K for 40 min.

The results of the elemental distribution of the original alloy samples and the specimen obtained under the optimum conditions are depicted in Figure 4. As can be seen in Figure 4, a uniform distribution of Ti, Al, and Cr elements is observed both before and after EML, while oxygen exhibits a radically different distribution. Figure 4a reveals that the elemental composition of the original alloy matrix predominantly comprises Ti and Al, with the presence of aggregated black striped substances on the surface. As seen in the SEM–EDS result for point S2 in Figure 4a, the Al and O element contents are determined to be 41.8 at.% and 57.6 at.%, respectively. Consequently, the inference drawn is that the black substance corresponds to Al_2_O_3_ [30]. As seen in Figure 4b, element surface scanning from the SEM image of the levitated sample was conducted, and a small amount of oxygen content was detected. The black striped substances were determined to be an oxide by the surface scanning results. Quantitative test by the oxygen/nitrogen analyzer revealed that the refined sample contained 0.258 at.% of oxygen elements, compared to 0.5 at.% in the initial sample. This indicates a significant reduction in oxygen content in the alloy after electromagnetic levitation melting. The EDS surface scan images show that the black striped substances are dissipated and become aggregated flakes and interconnections. EML resulted in the disappearance of microcracks on the sample’s surface, and the size and morphology of the black substance significantly improved. Combined with the thermodynamic calculations in the previous work, the impure oxygen notably decreased in levels and aggregated towards voids, and this may be the migration path of the oxygen removal process [31].

**Figure 5:**
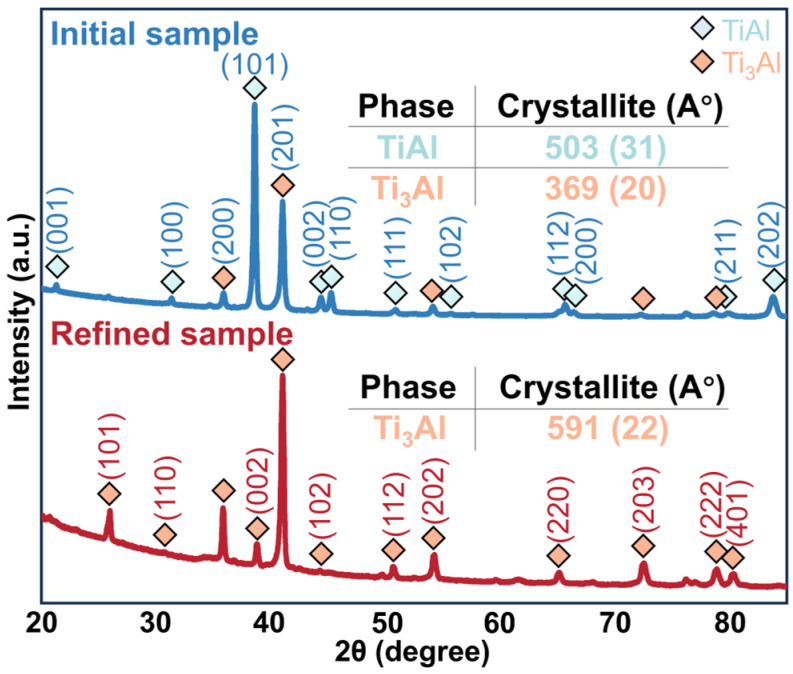
XRD images of the sample before and after EML.

The SEM–EDS analysis verifies significant alterations in the existence form, distribution, and composition of the oxygen element in the alloy after EML levitation. Meanwhile, the samples were subjected to an X-ray diffraction (XRD) analysis to investigate changes in their phase composition; the results are depicted in Figure 5. The initial sample exhibited crystalline peaks at diffraction angles (2θ) at approximately 31.7°, 38.7°, 44.5°, 45.4°, and 65.5°, corresponding to characteristic peaks of the tetragonal crystal system in TiAl (space group P4/mmm (No.#123)). The crystalline peaks observed at approximately 36.1°, 41.2°, and 54.1° are attributed to the hexagonal crystal structure of Ti_3_Al (space group P63/mmc (No.#194)). The main diffraction peaks were observed at angles of 38.7° for the γ-TiAl phase and 41.2° for the α_2_-Ti_3_Al phase; it can be indicated the phase composition of the initial sample is the TiAl and Ti phase [32]. The refined sample exhibited crystalline peaks at diffraction angles (2θ) at approximately 26.4°, 36.1°, 38.9°, 41.2°, and 54.1°, corresponding to hexagonal crystal structure of Ti_3_Al. This confirms the sample is composed solely of Ti_3_Al phase. As shown in Table 2, the lattice parameters and axial ratio of the γ-TiAl phase decrease after EML, along with a reduction in the plane spacing of the main peak. The c/a ratio of the alloy sample decreased from 1.4416 to 0.8034 after levitation; when the oxygen content is less than 0.5 at.%, the c/a ratio decreases with decreasing oxygen content.

### 3.4. Analysis Results of Element Migration during EML Deoxidation Process

**Figure 6:**
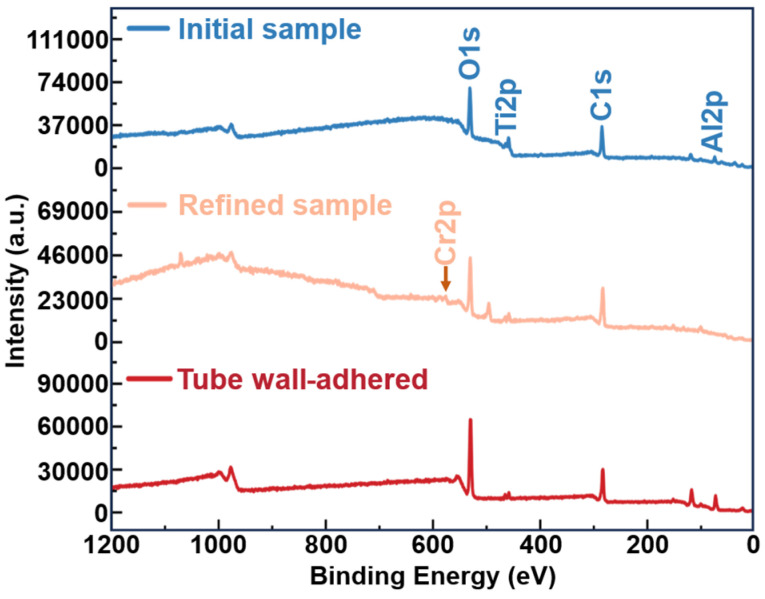
The XPS survey spectra of the substances under three different conditions.

X-ray photoelectron spectroscopy (XPS) was used to examine the surface characteristics of the alloy elements in the initial sample, the refined sample, and the residual volatile substance on the tube wall. The results were used to determine the binding states of oxygen with different alloy elements and to explore the migration pattern of oxygen elements. Figure 6 illustrates the XPS survey spectra of the initial, refined, and tube wall-adhered samples. In the XPS survey spectra under three different conditions, distinct O1s, Ti2p, and Al2p characteristic peaks are observed in the initial sample, indicating the presence of O, Ti, and Al as the main elements in the initial sample. In the levitated sample’s survey spectrum, besides the presence of O1s, Ti2p, C1s, and Al2p, characteristic peaks of Cr2p are also evident. Based on the XPS survey spectra of the three samples, the elements Cr, Al, Ti, and O are detected at around 576 eV, 73 eV, 459 eV, and 530 eV, respectively. By analyzing the fine spectra of C1s and performing charge correction using a binding energy of 284.8 eV, the binding states of each element in the levitated alloy are further clarified.

**Figure 7:**
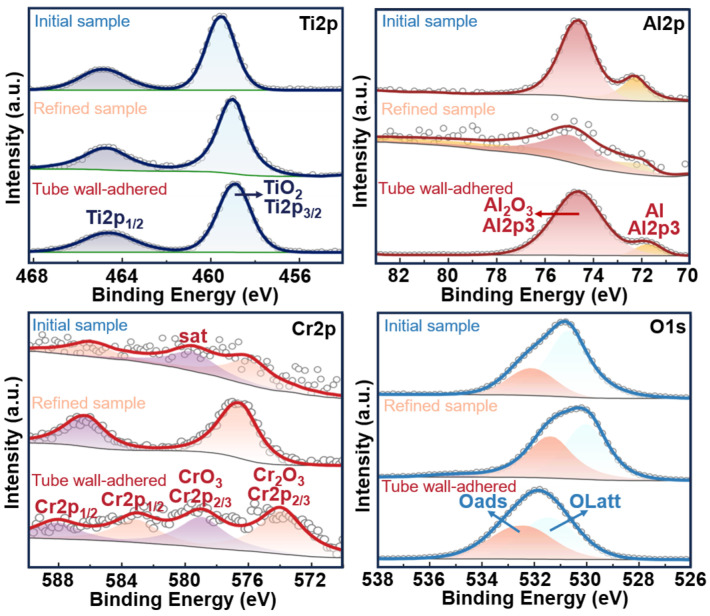
The XPS detection results for Ti, Al, Cr, and O elements.

Figure 7 presents the spectral analysis of Ti, Al, Cr, and O elements, and it reveals that in the XPS fine spectrum of Ti2p in the initial sample, two distinct peaks appear at 458.98 eV and 464.68 eV, corresponding to the spin–orbit split photoelectrons Ti2p3/2 and Ti2p1/2 of the Ti^4+^ chemical phase, respectively. This observation indicates the bonding of titanium with oxygen, attributed to Ti^4+^ within the TiO_2_ lattice. Similarly, in both the levitated and tube wall volatile substances, peaks corresponding to the spin–orbit split photoelectrons Ti2p3/2 and Ti2p1/2 of the Ti^4+^ chemical phase within the TiO_2_ lattice are observed at 458.88 eV, 464.58 eV and 458.86 eV, 464.56 eV, respectively. This indicates that the chemical state of titanium remains Ti^4+^ before and after the material processing. In the XPS fine spectrum of Al2p in the initial sample, two peaks appear at 72.28 eV and 74.58 eV, representing the characteristic XPS signals of Al (around 72 eV) and the binding peak of Al_2_O_3_. The proportions of these two peaks are 25.73% and 74.27%, respectively, indicating that Al mainly exists as Al_2_O_3_ in the initial sample, with some Al present as well. Similarly, peaks corresponding to Al and Al_2_O_3_ are detected in the levitated sample, with proportions of 37.79% and 62.21%, respectively. Compared to the initial sample, the relative content of Al in the levitated sample, in terms of Al, O, Ti, and Cr elements, is lower, decreasing from 32.88% to 12.24%. Furthermore, the overall spectrum confirms the lower total Al content in the refined sample, indicating a decrease in the content of both Al and Al_2_O_3_ after EML, which is due to the high vapor pressure of Al promoting the evaporation of Al_2_O_3_. In the tube wall volatile substances, peaks corresponding to Al and Al_2_O_3_ are also observed, with relative contents of 8.27% and 91.73% in the Al2p spectrum, indicating a higher content of Al_2_O_3_ adhering to the tube wall during the volatile process. Considering the low Cr content of only 1.0 at.% in the materials, the detected Cr content in both the initial sample and tube wall volatile substances is relatively low, while the peak intensity of Cr2p in the refined sample is higher, possibly due to the evaporation of other elements. In combination with the fine spectra, the relative content of Cr in terms of Al, O, Ti, and Cr elements is 3.04%, 10.47%, and 2.08%, respectively, and Cr exists in the form of Cr_2_O_3_ in all three materials.

In the fine spectra of O1s, oxygen elements are detected in the form of lattice oxygen (O Latt) and adsorbed oxygen (O ads) in both the initial, refined, and tube wall volatile samples. The relative elemental oxygen contents of each are 45.42%, 50.68%, and 42.8%, respectively. Furthermore, the relative contents of lattice oxygen are 32.58%, 25.99%, and 22.33%, respectively. The decrease in lattice oxygen corresponds to a decrease in the content of metal oxides on the alloy surface, while adsorbed oxygen can accelerate oxygen diffusion, thereby effectively improving the material’s electronic structure, geometric structure, and magnetic properties. To confirm the departure of lattice oxygen, this study conducted oxygen content detection before and after EML, revealing a decrease in oxygen content from 0.5 at.% to 0.29 at.%, indicating a reduction of 62% compared to before melting. This suggests that the high-temperature treatment of Ti-Al alloys using EML melting technology greatly promotes the migration of oxygen atoms.

**Figure 8:**
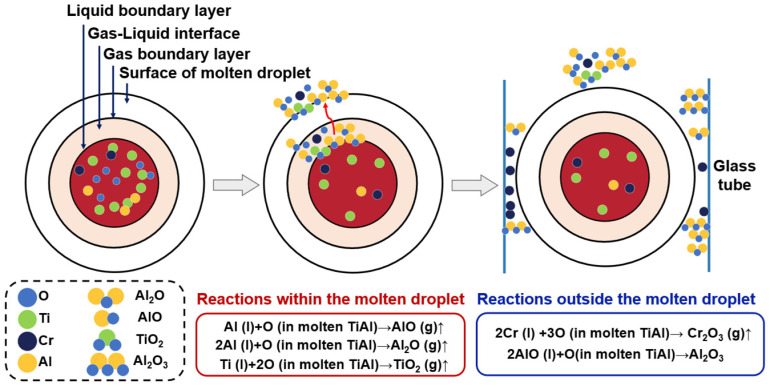
Deoxidation process schematic of Ti-Al alloys under EML.

As depicted in Figure 8, during the electromagnetic refining process of the Ti-Al alloy scrap, the migration modes of oxygen elements include the following three forms: (1) detachment from the crystal lattice to form AlO and Al_2_O and evaporation from the alloy melt; (2) formation of metal oxides remaining in the alloy melt; and (3) evaporation and adherence to the quartz tube wall as metal oxides.

The removal process of oxygen elements in Ti-Al alloys can be simplified into the following five steps:

(1) Oxygen atoms move into the melt boundary layer from the Ti-Al droplet interior.
(2) Oxygen atoms move through the melt boundary layer to the surface of Ti-Al droplets.
(3) Volatilization of oxygen atoms occurs at the gas–liquid interface.
(4) Oxygen atoms move through the gaseous boundary layer to the reaction chamber.
(5) Oxygen atoms condense on the glass wall or are extracted by a flowing gas system or vacuum system.

## 4. Conclusions

(1) The thermodynamic analysis of deoxidation indicated that oxygen in the Ti-Al system can be removed with the volatilization of aluminum, and the reaction is facilitated by increasing temperature. The deoxidation condition experiments revealed an optimal levitation temperature, time, and initial oxygen content of 2173 K, 40 min, and 0.5 at.%, resulting in an oxygen removal ratio of 62%.
(2) The impure oxygen in the alloy were significantly removed by EML, with a transition from a dispersed to a clustered distribution. The phase composition of the alloy changed from the TiAl and Ti_3_Al phases to a single Ti_3_Al phase. Furthermore, the lattice parameter c/a was reduced after EML.
(3) The refinement process of the Ti-48Al alloy scrap under EML can be divided into three forms of oxygen migration: (a) detachment from the crystal lattice to form AlO and Al_2_O, (b) formation of metal oxides remaining in the alloy melt, and (c) evaporation and adherence to the quartz tube wall as metal oxides.

## Figures and Tables

**Table 2:** The lattice parameters *a*, *c*, axial ratio (*c*/*a*), and interplanar spacing *d* of the sample before and after EML.

Alloy	a(Å)	c(Å)	*c*/*a*	d (Å)		
				(110)	(201)	(110)
Initial sample: TiAl	2.8228	4.0693	1.4416	2.3172	2.3253	2.0031
Initial sample: Ti_3_Al	5.7347	4.6327	0.8078			
Refined sample	5.7381	4.6101	0.8034	2.3070	2.1874	1.6906

## Data Availability

Data are contained within this article.

## References

[1] Güther V., Allen M., Klose J., Clemens H. (2018). Metallurgical processing of titanium aluminides on industrial scale. Intermetallics.

[2] Pere B.V., Joachim G., Andreas S., Norbert S., Jan H., Guillermo R. (2018). Peritectic titanium alloys for 3D printing. Nat. Commun..

[3] Mermer E., Çinici H., Uğur G., Ünal R. (2023). Development of an Al rich Ti-Al alloy with better ductility. Vacuum.

[4] Kim T., Oh J.-M., Cho G.-H., Chang H., Jang H.D., Lim J.-W. (2020). Surface and internal deoxidation behavior of titanium alloy powder deoxidized by Ca vapor: Comparison of the deoxidation capability of solid solution and intermetallic titanium alloys. Appl. Surf. Sci..

[5] Uwanyuze R.S., Kanyo J.E., Myrick S.F., Schafföner S. (2021). A review on alpha case formation and modeling of mass transfer during investment casting of titanium alloys. J. Alloys Compd..

[6] Chong Y., Gholizadeh R., Tsuru T., Zhang R., Inoue K., Gao W., Godfrey A., Mitsuhara M., Morris J., Minor A.M. (2023). Grain refinement in titanium prevents low temperature oxygen embrittlement. Nat. Commun..

[7] Reitz J., Lochbichler C., Friedrich B. (2011). Recycling of gamma titanium aluminide scrap from investment casting operations. Intermetallics.

[8] Kim T., Oh J.-M., Cho G.-H., Park J., Lim J.-W. (2020). Comparison of deoxidation capability of solid solution and intermetallic titanium alloy powders deoxidized by calcium vapor. J. Alloys Compd..

[9] Iizuka A., Ouchi T., Okabe T.H. (2022). New Deoxidation Method of Titanium Using Metal Filter in Molten Salt. Metall. Mater. Trans. B.

[10] Kim T., Lim J.W. (2023). Synthesis of low–oxygen Ti_3_AlC_2_ powders by hydrogenation–dehydrogenation and deoxidation from titanium scraps. J. Am. Ceram. Soc..

[11] Takeda O., Ouchi T., Okabe T.H. (2020). Recent progress in titanium extraction and recycling. Metall. Mater. Trans. B.

[12] Zhang Y., Fang Z.Z., Xia Y., Sun P., Van Devener B., Free M., Lefler H., Zheng S. (2017). Hydrogen assisted magnesiothermic reduction of TiO_2_. Chem. Eng. J..

[13] Suzuki R.O., Fukui S. (2004). Reduction of TiO_2_ in molten CaCl_2_ by Ca deposited during CaO electrolysis. Mater. Trans..

[14] Reddy R.G., Shinde P.S., Liu A. (2021). Review—The Emerging Technologies for Producing Low-Cost Titanium. J. Electrochem. Soc..

[15] Dring K., Dashwood R., Inman D. (2005). Predominance diagrams for electrochemical reduction of titanium oxides in molten CaCl_2_. J. Electrochem. Soc..

[16] Su Y., Wang L., Luo L., Jiang X., Guo J., Fu H. (2009). Deoxidation of titanium alloy using hydrogen. Int. J. Hydrogen Energy.

[17] Gökelma M., Celik D., Tazegul O., Cimenoglu H., Friedrich B. (2018). Characteristics of Ti6Al4V powders recycled from turnings via the HDH technique. Metals.

[18] Wang L., Su Y., Wang S., Luo L., Fu H. (2011). Effect of melt hydrogenation on structure and hardness of TC_21_ alloy. Rare. Metal Mat. Eng..

[19] Bartosinski M., Hassan-Pour S., Friedrich B., Ratiev S., Ryabtsev A. (2016). Deoxidation Limits of Titanium Alloys during Pressure Electro Slag Remelting. Mater. Sci. Eng. Conf. Ser..

[20] Cheng C., Dou Z.H., Zhang T.A., Zhang H.J., Su J.M. (2017). Synthesis of As-Cast Ti-Al-V Alloy from Titanium-Rich Material by Thermite Reduction. JOM.

[21] Brillo J., Egry I., Novakovic R. (2011). Surface tension of liquid Cu-Ti binary alloys measured by electromagnetic levitation and thermodynamic modelling. Appl. Surf. Sci..

[22] Yan P., Zhang G., Yi B., Mclean A. (2023). Kinetic characterization of phosphorus removal from the surface of metallurgical grade (MG) silicon droplets during electromagnetic levitation. Int. J. Heat Mass Trans..

[23] Vache N., Cadoret Y., Dod B., Monceau D. (2021). Modeling the oxidation kinetics of titanium alloys: Review, method and application to Ti-64 and Ti-6242s alloys. Corros. Sci..

[24] Brandes E.A., Brook G.B. (1983). Smithells Metals Reference Book.

[25] Baehr H.D. (1992). Thermochemical Properties of Inorganic Substances.

[26] Ono K., Moriyama J. (1982). Deoxidation of high-melting-point metals and alloys in vacuum. Metall. Mater. Trans. B.

[27] Oh J.-M., Seo J.-H., Lim J.-W. (2019). Refining effect of TiAl intermetallic compounds prepared by hydrogen plasma arc melting from scraps of Ti–Al mixture. Jpn. J. Appl. Phys..

[28] Chase M.W. (1998). NIST-JANAF thermochemical tables. J. Phys. Chem. Ref. Data.

[29] Sabat K.C., Murphy A.B. (2017). Hydrogen plasma processing of iron ore. Metall. Mater. Trans. B.

[30] Song Y., Dou Z., Zhang T., Liu Y. (2019). A novel continuous and controllable method for fabrication of as-cast TiAl alloy. J. Alloys Compd..

[31] Dong G., You X., Xu Z., Wang Y., Tan Y. (2022). A new model for studing the evaporation behavior of alloy elements in DD98M alloy during electron beam smelting. Vacuum.

[32] Schuster J.C., Palm M. (2006). Reassessment of the binary Aluminum-Titanium phase diagram. J. Phase Equilib. Diff..

